# Amyloid Core Formed of Full-Length Recombinant Mouse Prion Protein Involves Sequence 127–143 but Not Sequence 107–126

**DOI:** 10.1371/journal.pone.0067967

**Published:** 2013-07-03

**Authors:** Biswanath Chatterjee, Chung-Yu Lee, Chen Lin, Eric H.-L. Chen, Chao-Li Huang, Chien-Chih Yang, Rita P.-Y. Chen

**Affiliations:** 1 Institute of Biological Chemistry, Academia Sinica, Taipei, Taiwan; 2 Institute of Biochemical Sciences, National Taiwan University, Taipei, Taiwan; 3 Department of Biochemical Science and Technology, National Taiwan University, Taipei, Taiwan; University of Nebraska Medical Center, United States of America

## Abstract

The principal event underlying the development of prion disease is the conversion of soluble cellular prion protein (PrP^C^) into its disease-causing isoform, PrP^Sc^. This conversion is associated with a marked change in secondary structure from predominantly α-helical to a high β-sheet content, ultimately leading to the formation of aggregates consisting of ordered fibrillar assemblies referred to as amyloid. *In vitro*, recombinant prion proteins and short prion peptides from various species have been shown to form amyloid under various conditions and it has been proposed that, theoretically, any protein and peptide could form amyloid under appropriate conditions. To identify the peptide segment involved in the amyloid core formed from recombinant full-length mouse prion protein mPrP(23–230), we carried out seed-induced amyloid formation from recombinant prion protein in the presence of seeds generated from the short prion peptides mPrP(107–143), mPrP(107–126), and mPrP(127–143). Our results showed that the amyloid fibrils formed from mPrP(107–143) and mPrP(127–143), but not those formed from mPrP(107–126), were able to seed the amyloidogenesis of mPrP(23–230), showing that the segment residing in sequence 127–143 was used to form the amyloid core in the fibrillization of mPrP(23–230).

## Introduction

Transmissible spongiform encephalopathies (TSEs) are a group of fatal neurodegenerative disorders which affect many mammalian species such as human, bovine, sheep, deer, mink, cat etc [1]–[4]. The principle pathogenic event in TSE is the formation of an abnormally folded isoform, known as PrP^Sc^ from a normal cellular protein called PrP^C^ [5]. PrP^C^ is an evolutionary conserved glycosylphosphatidylinositol (GPI)-anchored glycoprotein of ∼254 amino acids and has been characterized structurally as having an unstructured N-terminal part and a C-terminal globular domain consisting of three α-helices and two short β-strands, together with one disulfide bond formed between helix 2 and 3 [6], [7]. Little is known about the exact structure of PrP^Sc^, except that it has high cross-β structure content [8]. As estimated by circular dichroism spectroscopy and Fourier transformed infrared spectroscopy, PrP^Sc^ has a β-sheet content of 43% compared to the 4% β-sheet content of PrP^C^, estimated from its NMR structure [9]–[11]. The conversion of PrP^C^ to PrP^Sc^ leads to a conformational change associated with an increase in β-sheet content [12], [13]. The characteristic features of PrP^Sc^ include its partial resistance to proteinase-K (PK) degradation, insolubility in non-ionic detergents, and a fibrillar structure typical of amyloid. After protease digestion, the protease-resistant core of PrP^Sc^ which consists of residues 90–231, has a molecular mass of 27–30 kDa and is therefore denoted as PrP 27–30 [14]. PrP^Sc^ has the ability to induce misfolding of PrP^C^, leading to the maintenance, propagation, and manifestation of disease phenotype in the host organism, with the result that, unlike other neurodegenerative disorders, prion diseases are transmissible.

The region of PrP^C^, which participates in the formation of PrP^Sc^, has long been a subject of interest, since it might provide information useful in designing inhibitors to interfere with the structural conversion of PrP^C^ and/or PrP^Sc^ propagation. Synthetic prion peptides of hamster origin covering residues 109–122 together with another overlapping 15-residue sequence 113–127 were found to form amyloids [15]. A chimeric mouse-hamster PrP of 106 amino acids (PrP106) with two deletions (Δ23–88 andΔ141–176) was found to retain the ability to support PrP^Sc^ formation in transgenic mice [16]. In addition, many short prion protein segments have been reported to have the ability to form amyloid fibrils. Synthetic prion peptides consisting of residues 106–126, 127–143, 106–147, 90–145, 171–193 (helix 2), or 199–226 (helix 3) has been found to form amyloid fibrils *in vitro* [17]–[21]. Human PrP(106–126) monomer has been reported to induce apoptosis in cultured neurons, and oligomers of this peptide have been shown to form cation channels and disrupt a model membrane non-specifically [22]–[27]. The importance of segment 106–147 was further established by the study of the crystal structure of the PrP globular domain, consisting of residues 123–230, which showed that the first β-strand (residues 128–131) of PrP^C^ forms an intramolecular β-sheet with residues 161–163 [28]. Molecular dynamics simulations showed that all three helices were conserved during the conversion of PrP^C^ to PrP^Sc^, while an additional β-strand was predicted to form in the loop between β-strand 1 (residues 128–131) and helix 1(residues 144–154) [29], [30]. Moreover, it has been reported that a lipid-anchored 61–amino acid peptide from prion protein (lacking residues 23 to 88 and 141 to 221 of the mature prion protein), termed PrP61, can form β-sheet-rich protease-resistant fibrils at a physiological pH [31]. Therefore the region encompassing the first β-strand and the loop connecting the first β-strand to helix 1 might participate in the structural conversion and amyloid fibril formation.

Since amyloid formation is one of the hallmarks of prion pathogenesis, it is an excellent *in vitro* model for studying the critical region involved in the structural conversion. Considering the nucleation-dependent polymerization model of amyloidogenesis, the ability of synthetic peptide-derived amyloid fibrils to act as nuclei for the polymerization of full-length PrP would shed light upon the relative importance of different regions as cores for PrP amyloid formation. In this study, three synthetic peptides, mPrP(107–143), mPrP(107–126), and mPrP(127–143), were synthesized and the amyloid fibrils formed from these three peptides were used as seeds to determine the segment within sequence 107–143 which can act as the core region in prion protein amyloidogenesis *in vitro*, based on the ability of these peptides to cross-seed full-length prion protein mPrP(23–230).

## Materials and Methods

### Peptide Synthesis

The prion peptides used were synthesized using the Fmoc-polyamide method on a PS3 peptide synthesizer (Rainin, USA) [32]. The N- and C-terminal ends of the peptides were acetylated and amidated, respectively, in order to mimic the polypeptide bond in the full-length protein. The peptides were characterized by mass spectrometry after purification. After lyophilization, the peptides were stored at –30°C.

### Expression and Purification of Full-length mPrP(23–230)

For expression and purification of mPrP(23–230), we followed the protocol described by Makarava et al [33]. Briefly, pET101/D-TOPO-mPrP(23–230), a kind gift from Dr. Ilia V. Baskakov (Center for Biomedical Engineering and Technology, University of Maryland Biotechnology Institute, USA), was transformed into *Escherichia coli* strain BL21 Star (DE3) (Invitrogen, Carlsbad, California, U.S.A). After induction in the presence of 1 mM IPTG for 5 hr, the cells were harvested, suspended in cell lysis buffer (50 mM Tris-HCl, 100 mM NaCl, 1 mM EDTA, pH 8.0), and subjected to repeated freeze-thaw cycles. Unless stated otherwise, all subsequent steps were conducted at room temperature. The lysate was incubated with 0.2 mg/mL of lysozyme and 0.1 mM PMSF for 40 min with continuous stirring, then 1 mg/mL of deoxycholic acid was added and the mixture was incubated for 45 min, followed by addition of 5 µg/mL of DNase I and further 45 min incubation. Inclusion bodies were harvested by centrifugation of the lysate at 12,000g for 30 min at 4°C and solubilized in buffer A (8 M urea, 0.1 M Na_2_HPO_4_, 10 mM Tris-HCl, 10 mM reduced glutathione, pH 8.0). After centrifugation at 20000g for 30 minutes at 4°C, the soluble fraction was loaded onto a prepacked Ni column (HisTrapTM ^FF^ 1 mL, Amersham Biosciences) previously equilibrated with buffer A and non-bound proteins were removed by washing. Then mPrP(23–230) was eluted with buffer A at pH 4.5. The eluted protein was desalted on a HiPrep™ 26/10 desalting column (Amersham Biosciences) at room temperature using 6 M urea, 0.1 M Tris-HCl, pH 7.5, as desalting buffer. Disulfide bond formation of the prion protein was induced by overnight oxidation at room temperature in the presence of 0.2 mM oxidized glutathione and 5 mM EDTA. The oxidized protein was purified at room temperature by reverse-phase chromatography on a C5 column (Discovery BIO Wide Pore C5, 10 µm, 25 cm×10.0 mm, Supelco, USA) with a 30 min linear gradient of 28–43% of buffer B (acetonitrile containing 0.1% trifluoroacetic acid). Oxidized mPrP(23–230) was eluted at about 33.3% of buffer B. The eluted protein was lyophilized and identified by ESI-TOF mass spectrometry and SDS-PAGE and stored at −30°C.

### Thioflavin T (ThT) Binding Assay

Amyloid fibril formation of spontaneous and seeded amyloidogenesis of mPrP(23–230) was monitored using the Thioflavin T (ThT) binding assay [34]. Briefly, 30 µL of 200 µM ThT in 140 mM NaCl, 100 mM phosphate buffer (pH 8.5) was mixed with 30 µL of fibril solution for 1 min at room temperature and the fluorescence emission between 460 and 600 nm was measured in a 3-mm path-length rectangular cuvette in a FP-750 spectrofluorometer (JASCO, Japan) with excitation at 442 nm. Both excitation and emission slits were set at 5 nm.

### Spontaneous Amyloid Fibril Formation by Mouse Prion Protein and Peptides

Purified recombinant mPrP(23–230) was dissolved in 6 M guanidine hydrochloride (GdnHCl) as a 132 µM stock solution. For fibrillization, 100 µL of the stock solution was diluted to 22 µM in 300 µL of fibril formation buffer (2x phosphate-buffered saline (PBS), 6 M urea, pH 6.0) and 200 µL of de-ionized water to give a final buffer composition of 1 M GdnHCl, 3 M urea in 1XPBS, pH 6.0 and the mixture was incubated 37°C with vigorous shaking (around 200–250 r.p.m.).

Peptides mPrP(107–143) and mPrP(127–143) were dissolved in deionized water as 100 µM stock solutions. The kinetics of amyloid formation was monitored in SpectraMax Gemini EM (Molecular Devices). Samples containing 50 µM of peptides in presence of 140 mM NaCl and 20 mM NaOAc, pH 3.7 and 10 µM ThT were incubated in 96 well assay plate (Corning, NY) at 25°C without shaking and kinetics was monitored by bottom reading of fluorescence intensity at every three hours interval using 445 nm excitation and 487 nm emission.

Peptide mPrP(107–126) was dissolved at a concentration of 754 µM in 20 mM HEPES buffer, pH 7.4, 100 mM NaCl, 0.01% NaN_3_ and dissolution was assisted by sonication. The kinetics was measured at 37°C using SpectraMax Gemini EM as described above.

All set of experiments were measured in triplicate and subsequent results were expressed as average.

### Seed Preparation for the Seeding Assay

Amyloid fibrils, generated as described above, were spun down and re-suspended in de-ionized water. The concentration of monomer remaining in solution after the above centrifugation was determined by UV absorption, except for mPrP(107–126), which does not contain Tyr, where the monomer concentration in solution was determined by HPLC. Fibrils generated from peptides or from full-length protein were fragmented using, respectively, 20 or 60 cycles of intermittent pulses (one cycle consists of five pulses of 0.6 sec with a 5 sec interval between two consecutive cycles) with an ultrasonic processor (UP100H, Hielscher, USA) equipped with a 1 mm microtip immersed in the sample. The power during operation was set at 40%. The length of the fragmented fibrils was around 200 nm [34].

To prepare PK-digested mPrP(23–230) seed, the amyloid fibrils were spun down at 15,600 g for 30 minutes and digested for 1 h at 37°C with PK (enzyme to substrate ratio of 1∶50), then the digestion was terminated by addition of 5 mM PMSF, and the PK-digested fibrils were spun down and used for preparing seed as described above.

### Seeding Assay

For the seeding reaction, different amounts of sonicated seed were added to the monomer solution and amyloidogenesis was monitored by the ThT binding assay at different incubation times. The seed concentration could not be exactly determined because of the polymeric nature of the fibrils and was therefore expressed as the amount of protein or peptide monomer incorporated into amyloid fibrils. For example, in case of fibrillization of 1 mL of mPrP(23–230) solution (protein concentration 22.07 µM), 17.74 µM of monomer (17.74 nmoles of monomer) was recruited to amyloid fibrils whereas 4.33 µM of monomer (4.33 nmoles of monomer) remained in the supernatant after the amyloid fibrils were spun down. After centrifugation the fibrils were suspended in 200 µL of de-ionized water which gave rise to a concentration of 88.7 pmoles of mPrP(23–230) monomer per microliter fibril suspension. Likewise, in case of mPrP(107–143) and mPrP(127–143) after centrifugation the fibrils were suspended in 200 µL of de-ionized water which gave rise to 131.9 pmoles of mPrP(107–143) and 43.5 pmoles of mPrP(127–143) monomer per microliter fibril suspension. Lastly, 742.6 nmoles of mPrP(107–126) monomer was participated in fibril formation and the amyloid fibril was suspended in 200 µL of de-ionized water which gave rise to 1.8 nmoles of mPrP(107–126) monomer per microliter.

### Data Analysis

Kinetic parameters for aggregation were obtained by monitoring the ThT-amyloid fluorescence signal over time and fitting fluorescence (F) data points to the sigmoidal curve of the following equation using Origin 8.0 graphical software [35].
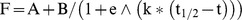



The above equation fits very well with fibril formation data. In this equation, A is the fluorescence during the lag phase, B the difference in fluorescence between the lag phase and post-transition plateau, t the incubation time, *t_1/2_* the time required to reach half the maximum ThT emission and k the rate constant of fibril growth (h^−1^). We defined the lag time as *t_1/2_–*2/k for each fitted curve.

### Transmission Electron Microscopy

Amyloid fibrils, generated as described above, were loaded onto carbon-coated 300-mesh copper grids, incubated for 4 min for absorption and then stained with 2% uranyl acetate for 4 min. After overnight drying in a desiccator, the samples were viewed on a Hitachi H-7000 electron microscope at 75 kV.

## Results

### Spontaneous Amyloid Fibril Formation by Mouse Full-length Prion Protein and Prion Peptides

**Figure 1:**
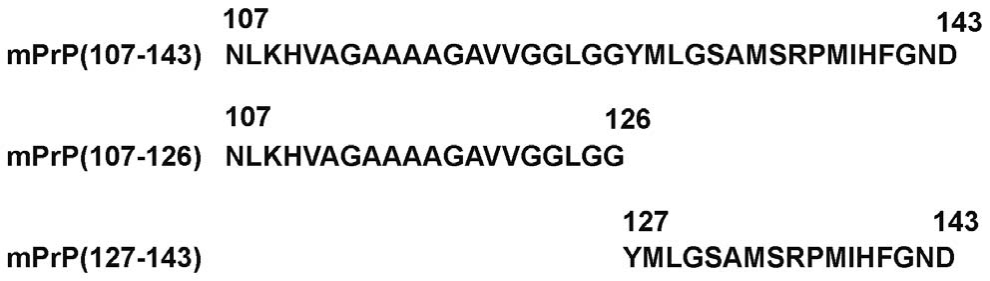
Amino acid sequences of the prion peptides used.

**Figure 2:**
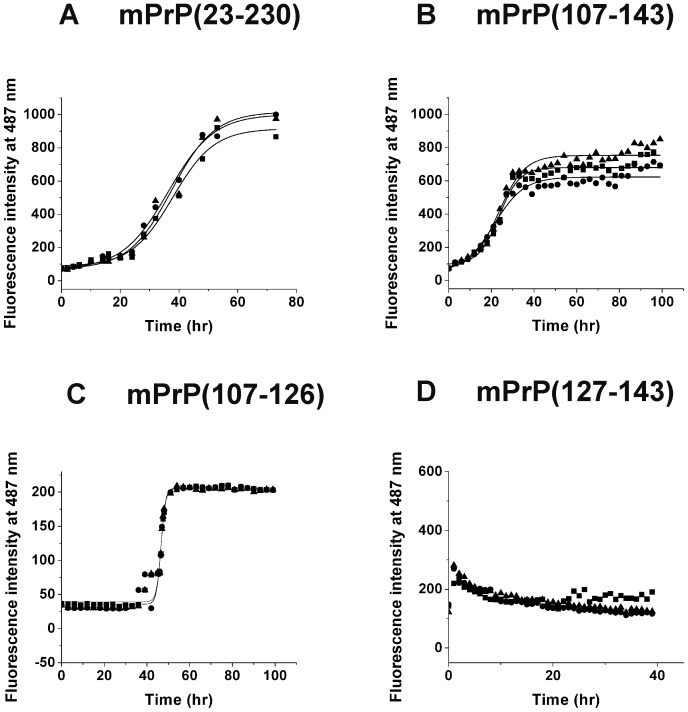
Spontaneous amyloid fibril formation of full length prion protein and prion peptides. Results from three independent measurements (denoted by closed square, ▪; closed circle, • and closed up triangle, ▴) are shown. (A) mPrP(23–230) (22 µM) in 1 M GdnHCl, 3 M urea in PBS, pH 6.0, was incubated at 37°C with shaking at 220 rpm. (B and D) mPrP(107–143) and mPrP(127–143) (50 µM), respectively in 140 mM NaCl and 20 mM NaOAc, pH 3.7, were incubated at 25°C without shaking. (C) mPrP(107–126) (754 µM) in 100 mM NaCl, 20 mM HEPES, pH 7.4, 0.01% NaN_3_, was incubated at 37°C without shaking. The kinetics of amyloidogenesis was monitored by ThT binding assay.

**Figure 3:**
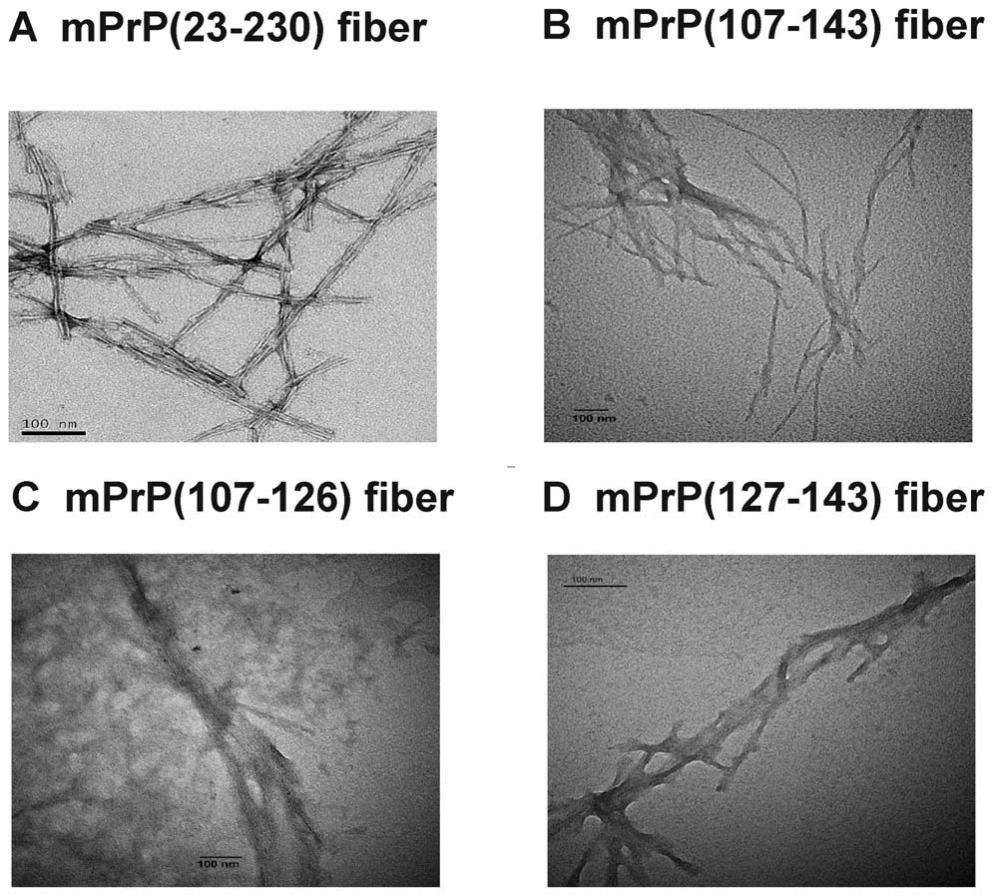
Morphology of amyloid fibrils. Transmission electron microscope images showing morphology of amyloid fibrils formed from (A) mPrP(23–230), (B) mPrP(107–143), (C) mPrP(107–126), or (D) mPrP(127–143). The bars represent 100 nm.

The sequences of the three prion peptides used are shown in Figure 1. Spontaneous amyloid fibril formation by full-length mouse prion protein mPrP(23–230) in 1X PBS containing 1 M GdnHCl and 3 M urea, pH 6.0; by mouse prion peptides mPrP(107–143) and mPrP(127–143) in 140 mM NaCl in 20 mM NaOAc, pH 3.7, and by mPrP(107–126) in 20 mM HEPES buffer, pH 7.4, containing 100 mM NaCl and 0.01% NaN_3_ was monitored by measuring the fluorescence emission of the amyloid fibril-ThT complex at 487 nm over time (Figure 2). For each experimental condition, three independent kinetic measurements were carried out. The typical sigmoid nature of the ThT-amyloid fluorescence plot indicates the generation of a molecular population with increased β-sheet content, an indicator of amyloid formation. In the case of the full-length protein mPrP(23–230), 1 M GdnHCl and 3 M urea were required to destabilize the native structure to facilitate its conversion into fibrils, and shaking was necessary to increase the chance of protein contact [36], [37]. The average lag time for spontaneous amyloidogenesis of mPrP(23–230) monomer in three independent experiments was 22.4 hours (Figure 2A). When peptides mPrP(107–143) and mPrP(127–143) were incubated in 140 mM NaCl and 20 mM NaOAc, pH 3.7, the average lag time for amyloidogenesis of mPrP(107–143) was 12.9 hours (Figure 2B), whereas mPrP(127–143) formed fibrils very rapidly and its lag time was hardly detectable (Figure 2D) suggesting mPrP(127–143) is much more amyloidogenic than mPrP(107–143). In contrast, mPrP(107–126) did not form fibrils under the same conditions (data not shown) and a very high peptide concentration (754 µM) and a different buffer (20 mM HEPES buffer, pH 7.4, 100 mM NaCl, 0.01% NaN_3_) were required for amyloidogenesis (Figure 2C) [38]. Under this condition the average lag time for spontaneous amyloidogenesis of mPrP(107–126) was found to be 41.2 hours. Figure 3 shows the morphology of these four kinds of fibrils under transmission electron microscope and that no significant difference in morphology was apparent.

### Cross-seeding Ability of mPrP(107–143) and mPrP(23–230)

**Figure 4:**
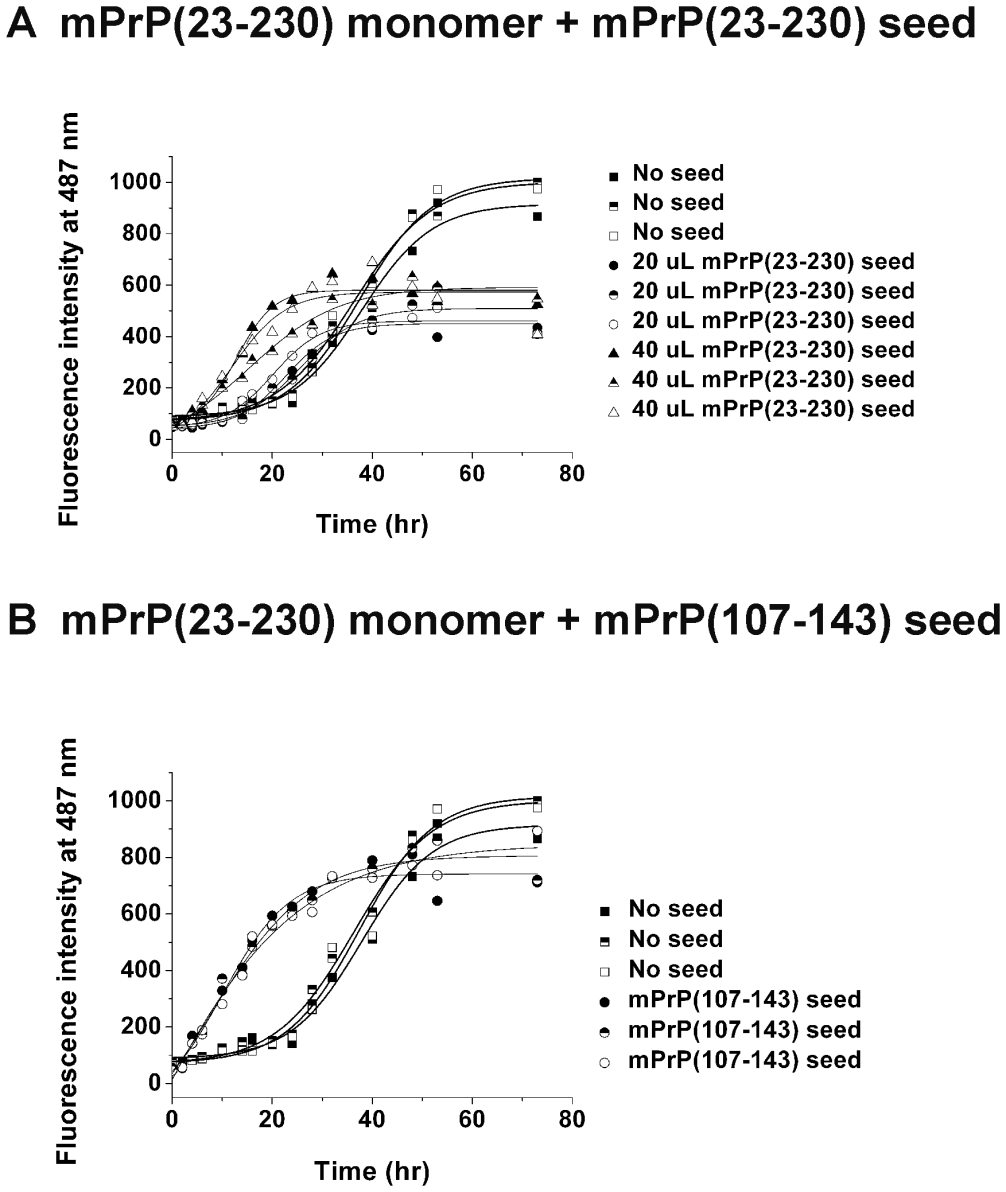
Amyloid fibril formation by mPrP(23–230) seeded with mPrP(23–230) seed or mPrP(107–143) seed. (A) Amyloidogenesis of mPrP(23–230) (22 µM) in 1 M GdnHCl, 3 M urea in PBS, pH 6.0, incubated at 37°C with vigorous shaking in the absence of seed (denoted by closed, half-filled and open squares for three independent measurements) or in the presence of 20 µL (denoted by closed, half-filled and open circles for three independent measurements) or 40 µL (denoted by closed, half-filled and open up triangles for three independent measurements) of sonicated mPrP(23–230) seed. In these three seeding experiments, mPrP(23–230) seed was prepared independently and three batches of seed contained 88.7, 88.3 and 96.2 pmoles of mPrP(23–230) monomer per microliter seed solution. (B) Amyloidogenesis of mPrP(23–230) under the same conditions as in (A) cross-seeded with 20 µL of sonicated mPrP(107–143) seed (denoted by closed, half-filled and open circles for three independent measurements). The seed contained 131.9, 129.1, and 119.7 pmoles of mPrP(107–143) per microliter solution in these three independent measurements. The data of spontaneous amyloidogenesis without seeding are also shown in the same plot for comparison.

If the amyloid fibrils of mPrP(107–143) and mPrP(23–230) use the same segment to form the cross-β structure, fibrils formed from mPrP(107–143) should be able to serve as template (seed) to initiate fibril formation by mPrP(23–230) monomer, and the fibrils formed from mPrP(23–230) should be able to initiate fibril formation by the mPrP(107–143) monomer. To prepare seed from a fibril suspension, centrifugation was first used to remove any remaining monomer, then ultrasonication was used to fragment long fibrils to generate seed [34]. In order to assess efficient fragmentation of fibril clump by sonication, we subjected the sonicated fibrils (seeds) to transmission electron microscopy as shown in Figure S1. Homologous seeding of the mPrP(23–230) monomer with the mPrP(23–230) seed showed a typical seeding effect in three independent experiments, as seen in Figure 4A where addition of 20 and 40 µL of mPrP(23–230) seed shortened the average lag time from 22.4 hours to 12.5 and 1.3 hours, respectively. On the other hand, heterologous seeding of mPrP(23–230) monomer with mPrP(107–143) seed showed immediate fibrillogenesis in all three independent measurements (Figure 4B).

**Figure 5:**
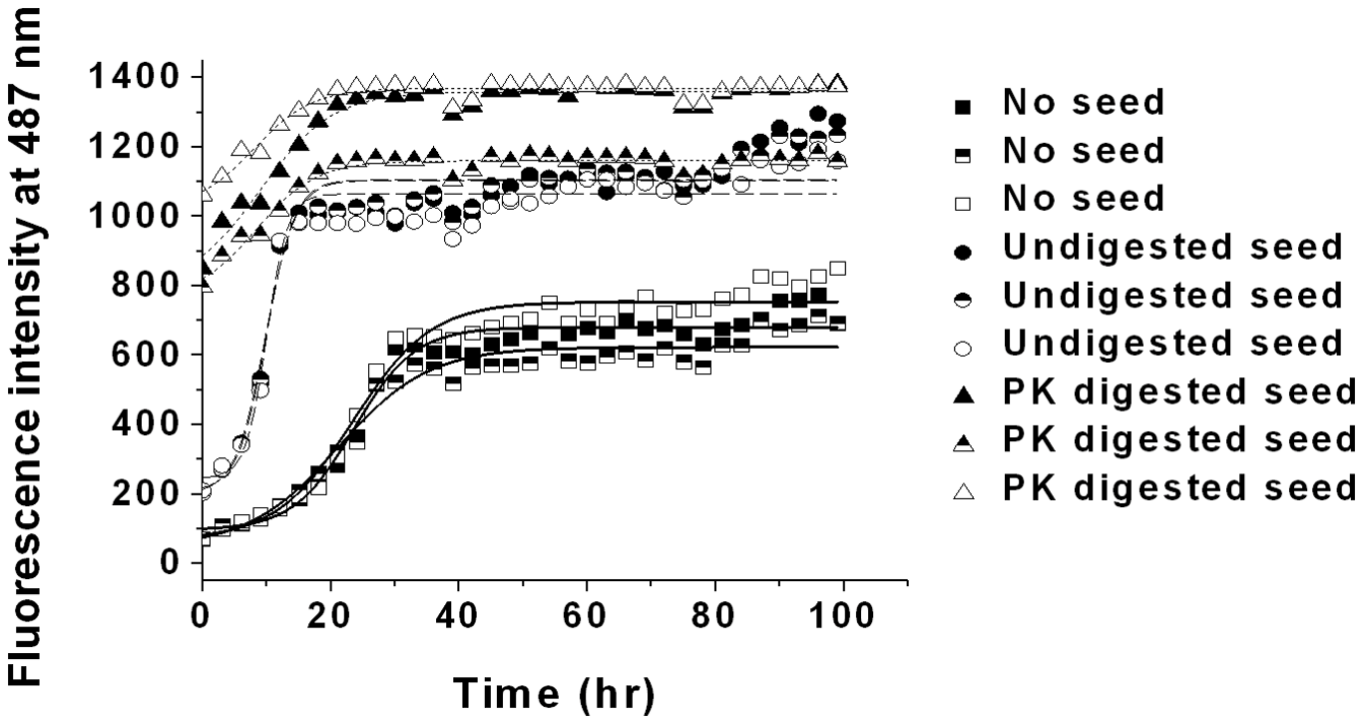
Amyloid fibril formation by mPrP(107–143) monomer in the absence of seed or in the presence of mPrP(23–230) seed with or without proteinase K digestion. mPrP(107–143) (50 µM) in 140 mM NaCl and 20 mM NaOAc, pH 3.7, was incubated at 25°C without shaking alone (denoted by closed, half-filled and open squares for three independent measurements), in the presence of 30 µL of mPrP(23–230) seed (denoted by closed, half-filled and open circles for three independent measurements), or in the presence of the same amount of mPrP(23–230) seed digested by proteinase K (denoted by closed, half-filled and open up triangles for three independent measurements).

We also examined the cross seeding effect of preformed mPrP(23–230) fibril on the amyloidogenesis of mPrP(107–143) monomer. As shown in Figure 5, addition of 20 µL of mPrP(23–230) seed shortened the average lag time from 12.9 to 5.2 hours. Because mPrP(23–230) is more than 5 times longer than mPrP(107–143), we wondered whether the presence of residues other than the amyloid core region might interfere the association between mPrP(107–143) monomer and mPrP(23–230) seed and affect seeding efficiency [39]. To test this, the same amount of mPrP(23–230) seed was digested with PK for 1 hr at 37°C to remove the non-amyloid part of the protein fibrils, then the PK-digested mPrP(23–230) fibrils were collected by centrifugation of the reaction mixture, re-suspended in the same volume of water (shown in Figure S2), and its seeding ability was tested. As shown in Figure 5, the PK-digested seed was apparently more effective at initiating amyloidogenesis of mPrP(107–143), indicating that the effective association between the amyloid core of the seed and the monomer is an important determinant of seeding efficiency.

### Comparison of the Cross-seeding Effect of mPrP(107–126) and mPrP(127–143) Fibrils on the mPrP(23–230) Monomer

**Figure 6:**
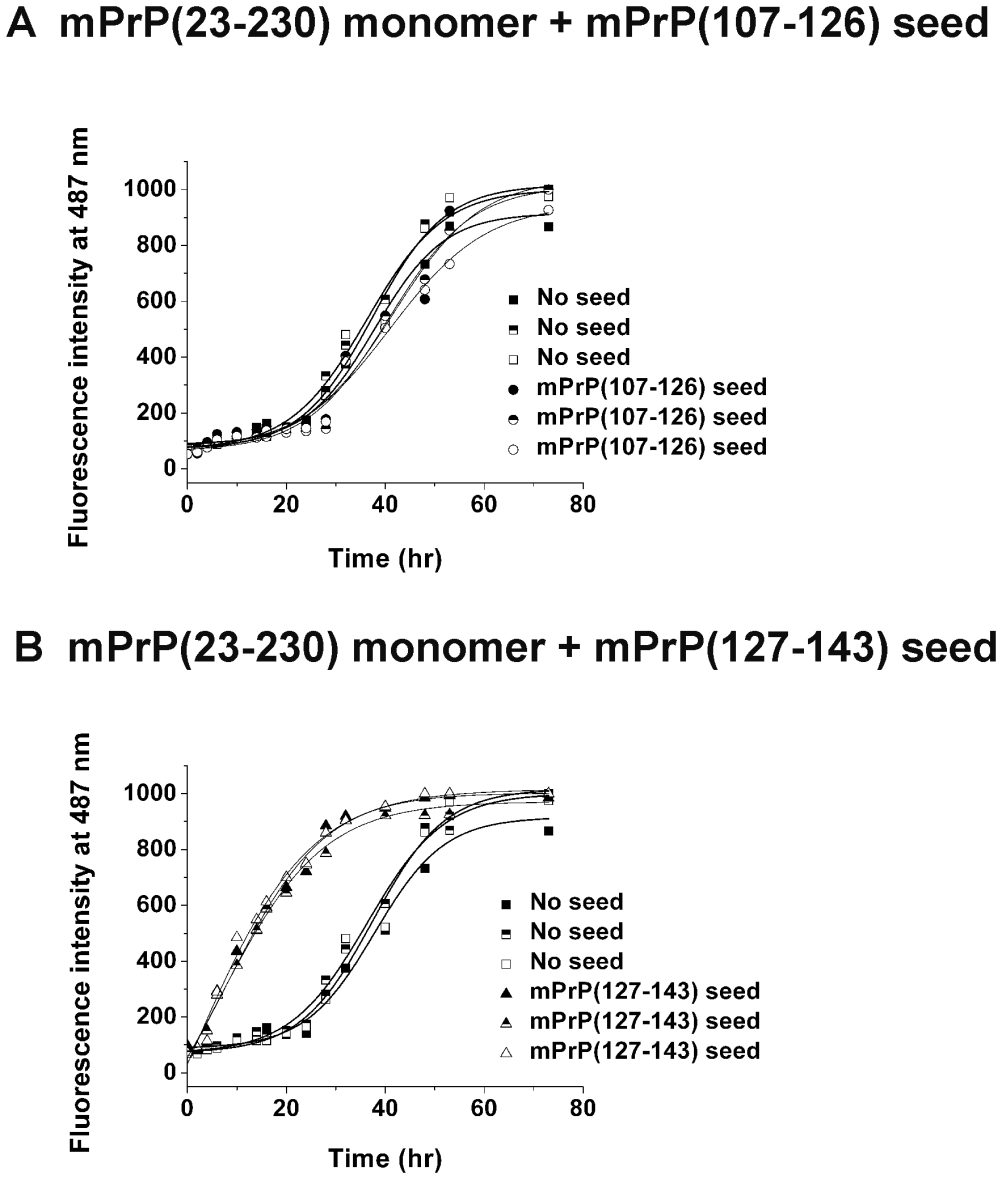
Amyloid fibril formation by mPrP(23–230) monomer cross-seeded with mPrP(107–126) seed or mPrP(127–143) seed. mPrP(23–230) (22 µM) in 1 M GdnHCl, 3 M urea in PBS, pH 6.0, was incubated at 37°C with vigorous shaking alone (in (A) as well as (B) denoted by closed, half-filled and open squares for three independent measurements) or in the presence of (A) 50 µL of mPrP(107–126) seed (denoted by closed, half-filled and open circles for three independent measurements) containing 1.8 nmoles of mPrP(107–126) per microliter or (B) 20 µL of mPrP(127–143) seed (denoted by closed, half-filled and open up triangles for three independent measurements) containing 43.5, 36 and 57 pmoles (for three independent measurements) of mPrP(127–143) monomer per microliter.

The results above suggested that mPrP(23–230) and mPrP(107–143) might share the same amyloid core. Within mPrP(107–143), the N-terminal half contains the amyloidogenic sequence “AGAAAAGA” and is more hydrophobic than the C-terminal half. However, as shown in Figures 2C and 2D, mPrP(127–143) formed amyloid rapidly and at a low concentration, whereas, in the case of mPrP(107–126), the amyloidogenesis was slow and required a very high peptide concentration. In the amyloidogenesis of the mPrP(23–230) monomer, shown in Figure 6, addition of 50 µL of mPrP(107–126) seed which contains as high as 1.8 nmoles mPrP(107–126) monomers per microliter seed solution still showed no seeding effect. In fact, the average lag time of amyloid formation of mPrP(23–230) was even slightly prolonged in the presence of mPrP(107–126) seed (Figure 6A), suggesting that mPrP(107–126) fibrils might interfere the self-association of mPrP(23–230). To test whether the lack of seeding effect of preformed mPrP(107–126) fibril was due to its less stability in the denaturing condition used for amyloidogenesis of mPrP(23–230) monomer throughout this study, we incubated all three fibrils generated from short prion peptides for 4 days in the fibrillization buffer and subjected to transmission electron microscopy to analyze morphology. As shown in Figure S3, all three peptide-generated fibrils remained intact under denaturing condition, ruling out the possibility of poor stability. In contrast, amyloidogenesis of mPrP(23–230) was induced immediately on addition of only 20 µL of sonicated mPrP(127–143) seed containing only 44 pmoles of mPrP(127–143) per microliter seed solution (Figure 6B), a seeding effect similar to that seen with mPrP(107–143) seed in Figure 4B. Our results showed that, though peptide mPrP(107–143) can seed full-length recombinant prion protein, the seeding ability resides in the C-terminal segment of this peptide.

## Discussion

The *in vitro* formation of amyloid fibril from soluble monomeric recombinant prion protein provides an insight into the structural conversion of prion protein, which ultimately leads to amyloidogenesis. With regard to the structure of soluble prion protein, it is important to locate the regions, which take part in the conversion process. According to several models, the process of β-aggregation starts when segments that possess high hydrophobicity, a high β-sheet propensity, and low net charge become exposed to the solvent and can associate [40]–[43]. Hydrophobicity analysis of the prion protein sequence revealed the existence of three hydrophobic clusters, one in the region of amino acids 110–137 and the other two reside in helices 2 and 3 [21]. The N-terminal half of mPrP(107–143), i.e. mPrP(107–126), formed spontaneous amyloid fibrils, though with a considerable lag phase. This is in agreement with the findings reported by Gasset *et al* [15]. One interesting point is that this peptide needed a much higher monomer concentration (754 µM) for initiation of fibril formation, but the monomer concentration remained in solution after fibrillization was only 12.4, 11.1 and 6.6 µM in three independent experiments. In contrast, the C-terminal half of mPrP(107–143), i.e. mPrP(127–143), underwent fibrillization without any detectable lag time for nucleus formation at a peptide concentration of 50 µM but the monomer concentration remained in solution after fibrillization was 32.6, 35.6 and 27.2 µM in three independent experiments. Our data suggested that (1) mPrP(127–143) might contain an intrinsic structural element that drives nucleation; (2) mPrP(127–143) has a higher thermodynamic solubility than mPrP(107–126); and (3) mPrP(107–126) might have a much higher energy barrier in the nucleation step. In this connection it is worth to mention that only having high hydrophobicity does not ensure a peptide segment of a protein to act as nucleation site where amyloidogenesis can begin. This notion is supported by the fact that in spite of having high hydrophobicity mPrP(107–126) requires high monomer concentration probably to overcome a high energy barrier during nucleation. In order to locate potential sites of nucleation which can act as amyloidogenic hot-spots we utilized two bioinformatic prediction methods, namely, FoldAmyloid [44] and Aggrescan [45], which use amino acid composition of proteins as the basic approach for assigning amyloidogenic hot-spots. Prediction from both the methods revealed potential amyloidogenic hot-spots within the sequence 127–143. Thus immediate fibrillization of prion peptide mPrP(127–143) might happen due to the presence of amyloidogenic hot-spots within this region that can act as a nucleation site.

While investigating the cross-seeding ability of mPrP(23–230) seed in the fibrillization of mPrP(107–143) monomer, the lag time was found to be shortened compared to the unseeded reaction, but not eliminated. This is in agreement with the ‘surface competition hypothesis’ that we proposed earlier [39]. Based on this hypothesis the process of seed induced fibrillization is a two steps phenomenon which involves initial ‘docking’ - the association of monomer with the preexisting seed followed by ‘locking’ i.e., the formation of cross-β structure between incoming monomer and the seed. In presence of protein segments not involved in the formation of amyloid core, the probability of incorrect binding between incoming monomer and seed becomes fairly high enough that leads to a detectable lag time for the reason that incorrect binding cannot go through subsequent elongation step resulting in a delay of fibril growth. Pre-digestion of the fibrils with PK reduces the probability of incorrect binding and favors the association between the monomer and the seeding nucleus thus shortening the lag phase greatly. Hence our data infer that the residues not involved in the structural conversion process could contribute to the species barrier in prion transmission [39]. Bocharova et al. reported that the PK-digested mPrP(23–230) fibrils harbor an epitope of a monocolonal antibody D18, part of which spans amino acid residues 133–143. This result also supports our conclusion that sequence 127–143 is in the PK resistance core of amyloid fibrils derived from mPrP(23–230) [46].

Cross-seeding of mPrP(23–230) monomer with mPrP(107–126) seed failed to shorten the lag time, even though a very high amount of mPrP(107–126) seed was used, indicating the inability of the full-length monomer to interact with the seed. The recruitment of a new monomer to a preexisting nucleus/fibril is an essential step in amyloid propagation. Successful cross-seeding relies on conformational adaptability between monomer and seed. Although, theoretically, all peptides or proteins have the potential to form amyloid structure and many prion peptides have been reported to be able to form amyloid fibrils, the lack of conformational adaptability between a specific monomer/seed pair might impede the successful propagation of prion amyloid formed from that particular monomer. Amyloid generated from mPrP(107–126) appears to be inaccessible to mPrP(23–230), accounting for the inability of mPrP(107–126)-derived fibrils to seed amyloidogenesis of mPrP(23–230) monomer. This has also been demonstrated by Kundu *et al* [47], who showed that amyloid fibril formed from synthetic PrP106–126 peptide (5%, wt/wt) was unable to seed 400 µM of huPrP23–144 monomer. In fact, in our cross-seeding experiment, the lag time for fibrillization with the mPrP(107–126) seed was even longer than in the unseeded reaction (Figure 6A). These results suggest that a longer time is required for the nucleation of the mPrP(23–230) monomer in the presence of mPrP(107–126) seed, i.e., the mPrP(107–126) seed inhibits the homologous association of mPrP(23–230) to form a nucleus. This is consistent with a previous report that hamster prion peptide P106–128 (corresponding to mouse sequence 105–127) has an inhibitory effect on the conversion of PrPsen (i.e. PrP^C^) to PrPres (PrP^Sc^) in a cell-free system [48].

The absence of a lag phase in the cross-seeding experiment using mPrP(23–230) monomer and fibrils derived from mPrP(127–143) suggests that the segment containing residues 127–143 probably forms the amyloid core of mPrP(23–230). Recently, Yamaguchi *et al.* [21] examined the amyloidogenic properties of several synthetic peptides and the ability of the peptide fibrils to induce amyloid formation of full-length mPrP and concluded that helix 2, and not helix 3, is important in initiating full-length mPrP fibrillization. Under their incubation conditions, only peptides corresponding to helix 2 and helix 3 could form amyloid fibrils, so only the fibrils derived from these two peptides were used in the cross-seeding reaction. In this regard it is essential to consider that a degree of structural cooperation might exist between sequence 127–143 and the C-terminal helices of prion protein in driving the structural conversion and the propagation of the converted structure. This argument is supported by a recent report showing that two sub-domains made up of [β-strand 1]-[helix 1]-[β-strand 2] and [helix 2]-[helix3] swap and separate from each other before dimerization [49]. Our results are also corroborated by the observation, made by Singh *et al.* [50], where they identified segments in prion protein, which exhibit multiple local conformations. They showed that the structural heterogeneity in the prion protein is not restricted to only the C-terminal domain rather it also manifests itself in the N-terminal segments like sequences 32–55 and 109–132 also. This study is highly pertinent to our work in a manner that both indicate to the contention that structural conversion of prion protein might be a collaborative phenomenon.

In conclusion, our study using seed-mediated *in vitro* amyloid formation shows that residues 127–143 harbor a critical region of prion protein, which, either independently or in conjunction with other critical regions, can initiate the seed-directed structural conversion of full-length prion protein.

## Supporting Information

Figure S1 **Fragmentation of preformed amyloid fibrils for seed preparation.** Preformed amyloid fibrils made from full length prion protein and prion peptides were collected by centrifugation at 15,600g for 30 minutes. The fibrils were suspended in water and sonicated as described in Materials and Methods. The sonicated fibrils were used as seed in seeding experiments. The sonicated fibrils (A) mPrP(23–230), (B) mPrP(107–143), (C) mPrP(107–126) and (D) mPrP(127–143) were viewed by transmission electron microscopy. The bars represent 100 nm.
(TIF)

Figure S2 **Proteinase K (PK) digestion of preformed amyloid fibrils made from mPrP(23–230).** Amyloid fibrils from spontaneous amyloidogenesis of mPrP(23–230) monomer were collected by centrifugation at 15,600g for 30 minutes at room temperature. The fibrils were then digested with PK as described in Materials and Methods. The PK-treated fibrils were viewed by transmission electron microscopy.
(TIF)

Figure S3 **Assessment of stability of amyloid fibrils made from short prion peptides in denaturing condition.** To assess the chemical stability of amyloid fibrils made from short prion peptides, preformed fibrils were incubated in the fibril formation buffer used for amyloidogenesis of mPrP(23–230) which contains 1 M GdnHCl and 3 M urea in PBS, pH 6.0 for 4 days at 37°C with shaking at 220 rpm. The integrity of fibril morphology (A) mPrP(107–143), (B) mPrP(107–126) and (C) mPrP(127–143) was analyzed by transmission electron microscopy.
(TIF)
