# Supplementary figures and images for: Amyloid Core Formed of Full-Length Recombinant Mouse Prion Protein Involves Sequence 127–143 but Not Sequence 107–126
Source: PLoS One. 2013 Jul 3;8(7):e67967. doi: 10.1371/journal.pone.0067967 (PMC3700907; doi:10.1371/journal.pone.0067967)

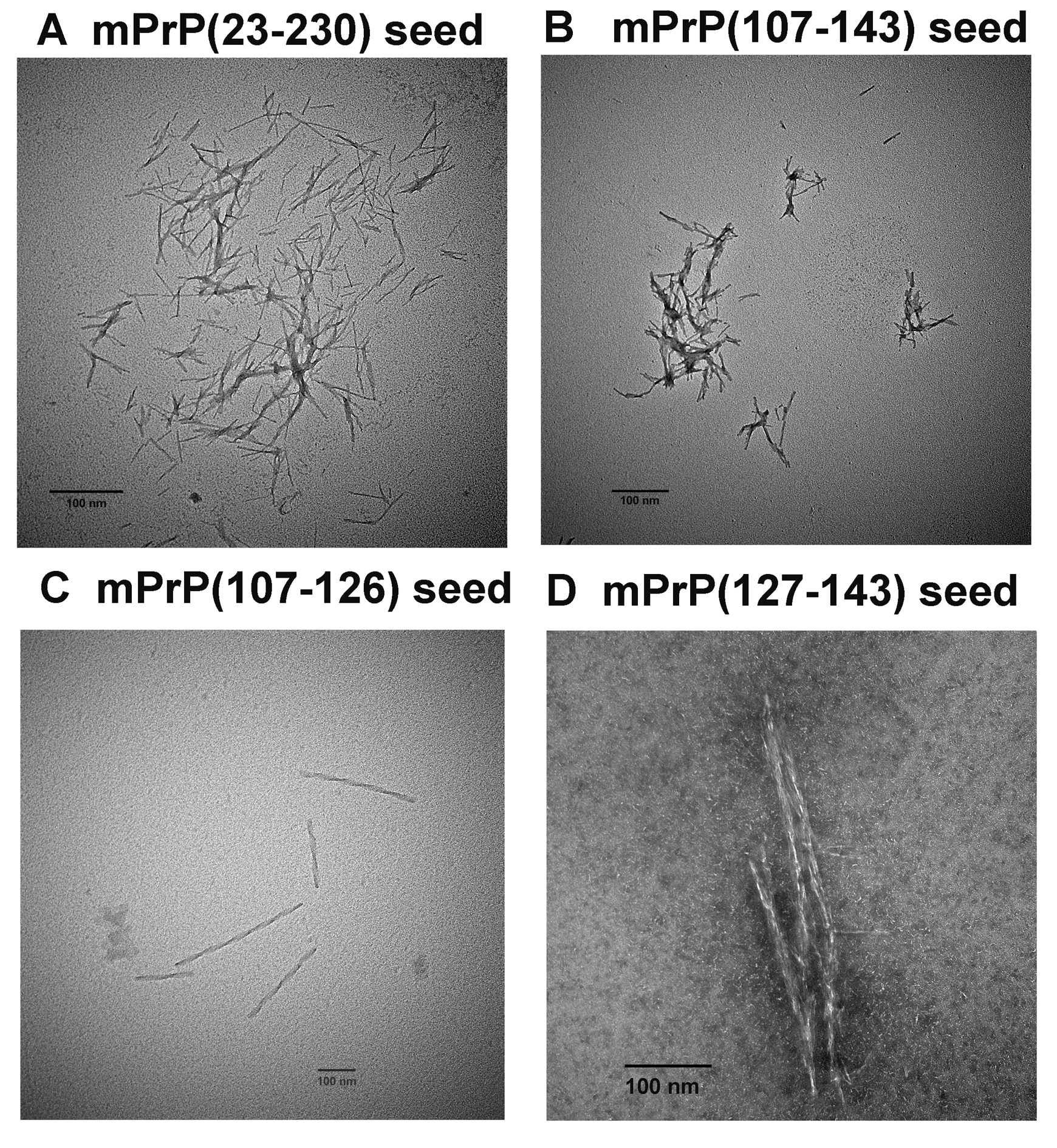

Supplement: Figure S1 — Fragmentation of preformed amyloid fibrils for seed preparation. Preformed amyloid fibrils made from full length prion protein and prion peptides were collected by centrifugation at 15,600g for 30 minutes. The fibrils were suspended in water and sonicated as described in Materials and Methods. The sonicated fibrils were used as seed in seeding experiments. The sonicated fibrils (A) mPrP(23–230), (B) mPrP(107–143), (C) mPrP(107–126) and (D) mPrP(127–143) were viewed by transmission electron microscopy. The bars represent 100 nm. (TIF) [file pone.0067967.s001.tif]

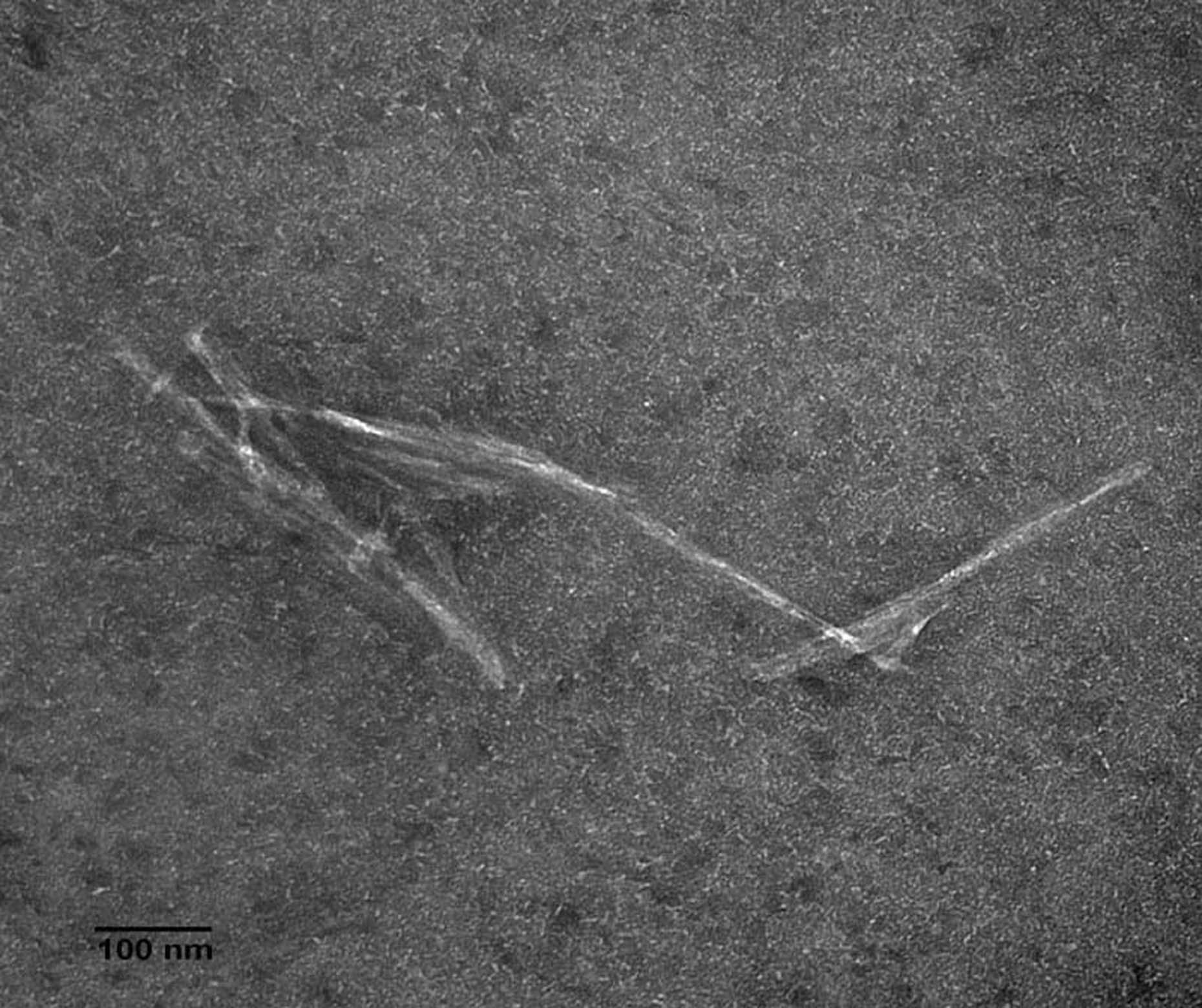

Supplement: Figure S2 — Proteinase K (PK) digestion of preformed amyloid fibrils made from mPrP(23–230). Amyloid fibrils from spontaneous amyloidogenesis of mPrP(23–230) monomer were collected by centrifugation at 15,600g for 30 minutes at room temperature. The fibrils were then digested with PK as described in Materials and Methods. The PK-treated fibrils were viewed by transmission electron microscopy. (TIF) [file pone.0067967.s002.tif]

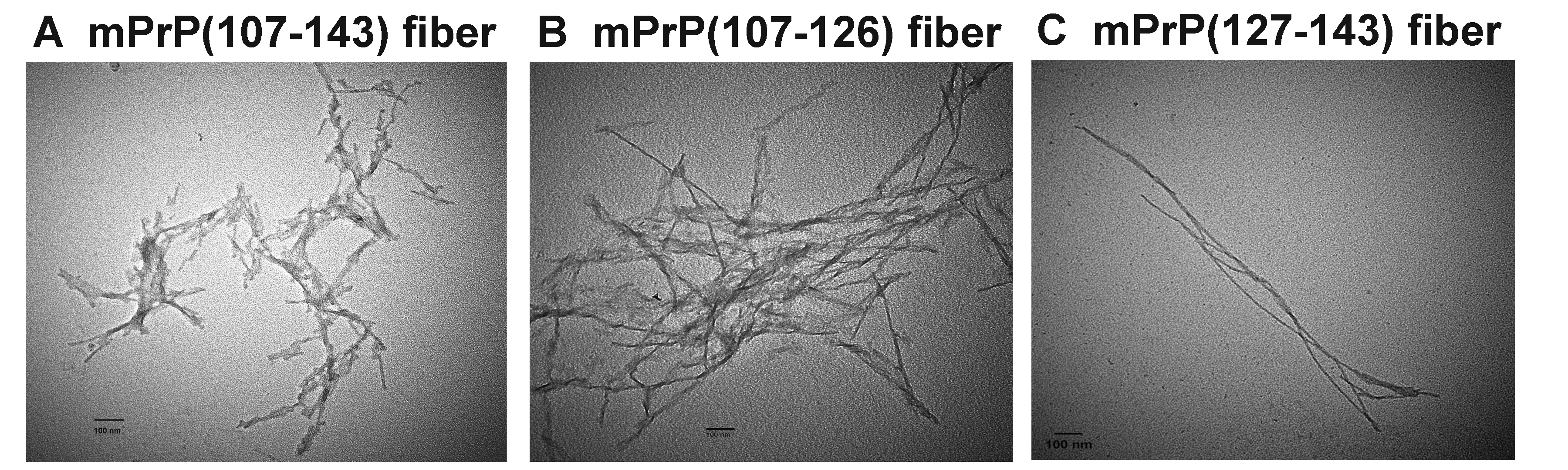

Supplement: Figure S3 — Assessment of stability of amyloid fibrils made from short prion peptides in denaturing condition. To assess the chemical stability of amyloid fibrils made from short prion peptides, preformed fibrils were incubated in the fibril formation buffer used for amyloidogenesis of mPrP(23–230) which contains 1 M GdnHCl and 3 M urea in PBS, pH 6.0 for 4 days at 37°C with shaking at 220 rpm. The integrity of fibril morphology (A) mPrP(107–143), (B) mPrP(107–126) and (C) mPrP(127–143) was analyzed by transmission electron microscopy. (TIF) [file pone.0067967.s003.tif]
